# Evaluation of Hexane Extract of Tuber of Root of *Cyperus rotundus* Linn (Cyperaceae) for Repellency against Mosquito Vectors

**DOI:** 10.1155/2009/908085

**Published:** 2009-10-13

**Authors:** S. P. Singh, K. Raghavendra, A. P. Dash

**Affiliations:** National Institute of Malaria Research (Indian Council of Medical Research), 22-Shamnath Marg, New Delhi 110054, India

## Abstract

Hexane extract of tuber of plant *Cyperus rotundus* (Cyperaceae) was screened under laboratory conditions for repellent activity against mosquito vector *Anopheles culicifacies* Giles species A (Diptera: Culicidae), *Anopheles stephensi* Liston (Diptera: Culicidae), and *Culex quinquefasciatus* Say (Diptera: Culicidae). The *Cyperus rotundus* tuber extract was used to determine their effect on mosquito vector, and comparison with the DEET (NN Diethyl 1-3 methyl Benzamide, formerly known as diethyl 1-m-toluamide). The tuber extracts showed more effective at all the dose. Result obtained from the laboratory experiment showed that the tuber extracts are more effective for repellency of allthe mosquito vector even at low dose. Clear dose response relationships were established with the highest dose of 10% tuber extract evoking 100% repellency. Percent protection obtained against *An. culicifacies* Giles species A 100% repellency in 4 hours, 6 hours, *An. stephensi* 100% repellency in 6 hours and *Cx. quinquefasciatus* was 100% repellency in 6 hours at the 10% concentration. Against DEET- 2.5% *An. culicifacies* A 100% repellency in 1 hour, 2 hours, 6 hours, An. *stephensi* have shown 100% repellency in 6 hours, and *Culex quinquefasciatus* have shown 100% repellency in 1 hour, 2 hours, 6 hours. The consolidated data of the repellency observed in different species is given and it is evident that the over all repellency rates varied between 80 and 100% for different repellents concentrations (2.5%, 5%, and 10%). The extract can be applied as an effective personal protective measure against mosquito bites.

## 1. Introduction

Malaria contributes the major disease burden and its control is hampered many operational and technical reasons and among the technical reason insecticidal resistance, namely, development of insecticide resistance in malaria vectors to the commonly used synthetic chemical insecticides in public health sprays has made the disease control more difficult [1]. Mosquito population has not been reduced significantly after continuous use of synthetic insecticides and *Anophelines* retain their potential to support the outbreak and development of malaria in endemic areas. *Anopheles stephensi* Liston and *Anopheles culicifacies* Giles are two major vectors of malaria in India and later is a sibling species complex. There are five sibling species present named A, B, C, D, and E, of which species A, C, D, and E are vectors while B is a poor vector [2]. *Culex quinquefasciatus* Say transmits Filariasis while dengue fever and DHF are transmitted by the vector *Aedes aegypti* Linn.

 In contrast to vaccines and chemoprophylaxis as means of personal protection, repellents are convenient, inexpensive, and afford advantages in protection against a wide range of vector [3]. They are also the primary means of mosquito-borne disease prevention available in areas where vector control is not practical [4, 5]. The majority of commercial repellent products contain the DEET (Diethyl 1-3 methyl Benzamide, formerly known as diethyl 1-m toluamide), which was first Synthesized in 1954 [6]. Although effective, Deet is not the ideal product, as allergic and toxic effects have been documented [4, 7] and its solvent characteristics can damage plastic and other synthetic materials. Deet, in combination with certain other agents, is suspected of causing Gulf War syndrome [8, 9], although this matter is still controversial. Because of the undesirable effect of Deet, research was actively carried out to find an alternative compound that is safer to use and equally or more effective (Robert et al. [10]), [11–13]. One promising new repellent is the piperidine compound, A13-37220, which provides equal or better protection against certain mosquitoes than that obtained with Deet [13–15]. Despite the significant repellency of A13-37220, there are concerns over its safety.

 The repellent potential of plant to mosquitoes and other pest insects has been well known both prior to [16] and after [17] the advent of synthetic chemicals, Various botanical substances, *Cymbopogan spp*. [18, 19], *Eucalyptus maculate citriodon* [20], *Azadirachta indica* [21], *Pelargonium citrosum* [22], *Lantana camara* [12], and *Mentha piperita* [23] have been reported as being repellent against adult mosquitoes. Some promising essential oils such as citronella. Lemon eucalyptus, neem, and peppermin*t* oils are derived from these plants and they are currently available in several commercially formulated repellents. However, their repellency is still lower in both efficacy and duration than that in currently used chemicals such as Deet and A13-37220. Nevertheless, the possible health risks associated with use of these chemicals should be taken in to consideration. With this in mind, the present study was carried out to investigate the repellent activity of the Indian medicinal plant, *Cyperus rotundus* against the mosquito *Anopheles culicifacies* Giles species A (Diptera: Culicidae)*, Anopheles stephensi* Liston (Diptera: Culicidae), and *Culex quinquefasciatus* Say (Diptera: Culicidae). This may provide a new and promising repellent that can be used in the development of an economical and practical means to protect humans from the mosquito bites. Several frequently used herbal drugs are derived from the *Cyperus rotundus* species of the family Cyperaceae. The tuber of the root of Cyperus is commonly used as traditional drugs. *Cyperus rotundus* has been traditionally used in India for food and medicinal activity such as antibacterial, analgesic, antipyretic, and antiplasmodic, antibacterial, analgesic, antipyretic, antiplasmodic (Glossary of Indian medicinal plant PID-Chopra et al. [24]) Presence of highly significant anti-inflammatory and antipyretic in *Cyperus rotundus *suggests a very useful remedy for arthritic condition. The presence of tranquilizing, hypotensive, smooth muscle relaxant, antiemetic, anthistaminic anti-inflammatory and antipyretic activities show that the extract possesses significant pharmacological activity [25].

## 2. Material and Methods

For these studies plants were collected from rural area of district Agra of Utter Pradesh and in Delhi state of north India. The plant was identified by the National Institute of Science Communication, The Wealth of India Division, Council of Scientific and Industrial Research, New Delhi, India.

### 2.1. Hexane Extracts of Tuber of Cyperus Rotundus

Fresh tuber was collected and washed with water, dried in shade, and powdered. The tuber of the root of *Cyoerus rotundus* powdered material of this plant (1 kg) was subjected to extract three times in a soxhlet apparatus using solvent of hexane in ratio of 1 : 3 (w/v) of 95% hexane at room temperature. The extract was made solvent free and the final residue of hexane extract of *Cyperus rotundus* obtained, lyophilized, and then kept at −20°C until testing for adult repellent activity. In preparing test concentrations, extract was volumetrically diluted in absolute hexane and Tween 80 at an appropriate test concentration.

### 2.2. Mosquito Strains

*An. culicifacies* species A, *An. stephensi*, and *Cx. quinquefasciatus* were used in this study. Mosquitoes were reared in a laboratory maintained at 27 ± 2°C temperatures and 70 ± 5% relative humidity with a photoperiod of 12 : 12 hours (Light : Dark) with 90 minutes down and dusk simulation periods. Adult's mosquitoes were provided with 10% sucrose. The 5–8 days old females used for investigation of repellent activity.

### 2.3. Mosquito Cages

One hundred-5–8 days old-sugar-fed female mosquitoes were introduced in to the cloth cages. These mosquitoes were starved for about 15 hours prior to their introduction in to the cages. A minimum of three replicates were prepared for a given species of mosquito. Needed standardization was made for experiments as to the determination of the suitable age of the mosquito for experiments and method of recording the data (Protocols for Uniform Evaluation of Insecticides for use in Vector Control. National Institute of Malaria Research—2005—http://www.mrcindia.org/).

### 2.4. Preparation of the Repellent and Control Replicates

500 mL of 10% sugar solution was prepared in water. Sufficient quantity of bleached cotton was taken to be stacked in to a 460 mL Styrofoam glass. 450 mL of the above sugar solution was poured into the glass and the cotton was soaked. The cotton at the top was stretched out side in to circular foam. Remaining 40 mL was used to prepare repellent formulation. To 40 mL of the sugar solution required quantity of the tuber hexane extract concentrate was mixed to arrive at the desired concentrations, namely, 2.5%, 5%, and 10% and was poured evenly on the sugar soaked cotton in the above Styrofoam glass. Similarly DEET 2.5% in 10% sugar soaked cotton was prepared for use as positive and only 10% sugar soaked cotton was used as negative controls, respectively. For tuber of root of *Cyperus rotundus* hexane extract, known quantity of residue extract was redissolved in hexane to make a 10% (w/v) stock solution. Various test concentrations ranging between 2.5%, 5%, and 10% were prepared in double distilled water using freshly made stock solution. Controls were supplemented with the equal amount hexane required for the experiment without extracts. Tween-80 was used as an emulsifier at 0.05% concentration in the final test solution.

## 3. Repellency Tests

Experimental cages with the mosquitoes were placed in this room. In these cages, the Styrofoam glasses with cotton soaked with three different concentrations of tuber hexane extract of *Cyperus rutundus* namely 2.5%, 5%, and 10% sugar solution, DEET 2.5% (positive control) in 10% sugar solution and 10% sugar solution (negative control) were placed in four different corners and one in the centre of the cage. Five-minute landing counts were made at 0, 1, 2, 4, 5, and 6 hours. The cups were removed from the cage after the five minute observation at each interval of time. The cup was covered to avoid evaporation of the insecticide formulation and was placed in the refrigerator. For subsequent exposure the position of the cups were inter changed to different corners (Protocols for Uniform Evaluation of Insecticides for use in Vector Control. National Institute of Malaria Research—2005—http://www.mrcindia.org/).

### 3.1. Data Analysis

Observation is made in at least three replicates for the given species of the mosquito that landed and attempted to feed were recorded. No mosquito landing occurred in the initial 3 minutes the cups interchanged into the different corners. Observations were made at 30 minutes intervals. If more than 1 mosquito landing was recorded during an observation, the test of repellency was terminated and the period of repellent protection calculated as the time between the extract application and multiple mosquito landing. The landing rates of the mosquitoes on different concentration of the formulation of hexane extract of tuber of root of Cyperus* rotundus* (2.5, 5, and 10 % (DEET 2.5% and sugar (10%) were recorded. Data was reported as mean of the observations for each of the formulation. Result was expressed as average landing counts per exposure interval and as repellency compared to control means using as follows [26]:

(1) $$\%\;\text{Repellency}\;=\frac{C-T}{C}\times 100,$$

where C: the mean number of landing on negative control (10% sugar solution); T: landing on the repellents (DEET and tuber of root of Cyperus rotundus).

## 4. Results

The consolidated data of the repellency observed in different species is given and it is evident that the overall repellency rates varied between 80–100% for different repellents, concentrations of repellents and species. The hexane extract of *Cyperus rotundus* was the candidate for subsequent field investigations repellent activity. Species and number of mosquitoes collected from the control are demonstrated in Table 1. A total of 100 adult mosquito comprising three species in two genera were rearing in laboratory NIMR. Table 1 summarizes the results of *Cyperus rotundus* laboratory testing, these results showed a highly significant difference between the mean landing counting on the treated and control. The hexane extract of tuber of the root seed showed strong repellent activity against adult mosquitoes. Percent protection obtained against *Anopheles culicifacies* Giles species A (100% in 4 hours, 6 hours)* Anopheles stephensi *(100% in 6 hours) and *Culex quinquefasciatus* were (100% in 6 hours) at the 10% concentration. Against DEET-2.5%* An. Culicifacies* A 100% repellency in 1 hour, 2 hours, 6 hours, An. *stephensi* have shown 100% repellency in 6 hours, and *Culex quinquefasciatus* have shown 100% repellency in 1 hour, 2 hours, 6 hours. However, testing hexane extract of *Cyperus rotundus* need to be carried out in the field. 

Hexane extract of *Cyperus rotundus* was found effective in repelling the three mosquito disease vectors*, Anopheles culicifacies* species A, *Anopheles stephensi* (malaria vectors), and *Culex quinquefasciatus* (filaria vector) though with minor variations. The percent repellency at different observation periods (0 hour, 1 hour, 2 hours, 4 hours, and 6 hours ranged from 80 to 100% against different concentrations, repellents, and species.

Against hexane extract of tuber of root of* Cyperus rotundus, Culex quinquefasciatus* has shown more than 90% repellency to all the concentrations (except 2.5% in 0 hour, 1 hour, 2 hours) that is, 2.5, 5, and 10%. *Anopheles culicifacies* species A and *Anopheles stephensi* have shown 90–100% repellency against at 5% and 10% concentrations, while at 2.5% the repellency ranged between 75–90%.

## 5. Discussion

In laboratory test, Cyperus* rotundus* hexane extract possessed significant repellent activity against the three species tested which is similar compared to that reported for currently used synthetic compounds such as DEET, A13-35765, A13-37220 and CIC-4 [14, 27]. These chemical compounds provide better and longer protection against many biting insects and level = 0.37–25.37 *μ*g/cm^2^, 2–8 hours). Repellent protection time in laboratory bioassays however can change depending on the biological characteristics of the mosquito test population. Differences in species and body size, sugar water availability, adult density in test cages, and mosquito age can affect test results [18, 28–30]. Tawatsin et al. [31] demonstrated under laboratory conditions that volatile oils derived from turmeric (*curcuma longa*), citronella grass (*Cymbopogon winterianus*), and hairy basil (*Ocimum americanum*) with the addition of 5% vanillin were effective in repelling both diurnal and nocturnal mosquitoes for up to six hours. The hexane extract of *Cyperus rotundus* was effective in reducing the vector species. When compared with the study of Sharma et al. [32], the protective effect against *An. Culicifacies, An. stephensi* and *Culex quinquefasciatus*, of hexane extracted of *Cyperus rotundus* seem to be higher than that of neem oil (37.5%). Selection of a repellent for further development cannot be based on the results of any one test against a single insect because mosquito responses to repellents vary within and among species [18, 33]. The protection *against Cx tritaeniorhynchus* and *Cx. quinquefasciatus*, the vectors of Japanese B encephalitis [34, 35] and filariasis [11, 36], respectively, is considered as satisfactory. The hexane-extracted *Cyperus rotundus* may protect against other mosquito vector species. The further studies should be investigated against as many different malaria vector as possible under both laboratory and field conditions. Several methods enhancing the efficacy of repellent, such as purification of the active fraction, increase in persistence and duration of repellency need to be studied. 

From the observed data on the repellency against the three disease vectors, it can be concluded that dose of 10% could be used for achieving the desired level of protection against bites of these mosquitoes. However, these results pertain to the effectiveness in cage experiments using only sugar solution as attractant. Thus, further confirmation by testing this repellent on human subjects to evaluate the repellency effect is needed. Our repellent research is being continued to search for the development of new repellents from a natural original that not only offer effective anti-mosquito products but are also biorational alternatives to synthetic pesticides. 

## Figures and Tables

**Table 1:** Percent repellency of hexane extract of tuber of root of *Cyperus rotundus Linn* against three important mosquito vectors.

Species Replicate	Doses %	Repellency in hours				
		0 hour	1 hour	2 hours	4 hours	6 hours
*An. culicifacies (3) *	Tre- 2.5	80	75	88	90	95
	Tre- 5	88	90	90	96	99.2
	Tre- 10	97	96	97	100	100
	Deet 2.5	98	100	100	99	100
*An. stephensi (3)*	Tre- 2.5	84	80	85.3	90	96
	Tre- 5	89	88.7	92.0	95	99
	Tre- 10	99.6	93.0	93.0	99	100
	Deet 2.5	96.7	100	90	99	100
*Cx. Quinquefasciatus (3)*	Tre- 2.5	83.5	75	82.3	91.7	95.8
	Tre- 5	89.2	89.7	93	95	99
	Tre- 10	89.6	96	95.5	99	100
	Deet 2.5	96.6	100	100	96.7	100

R- no of Replicate

N- no of mosquito
